# Influence of Protein Concentration on Heat-Induced Fouling of Oat Drink

**DOI:** 10.3390/foods15122248

**Published:** 2026-06-22

**Authors:** Phillip Müter, Vandita Verma, Jörg Hinrichs

**Affiliations:** Department of Soft Matter Science and Dairy Technology, Institute of Food Science and Biotechnology, University of Hohenheim, Garbenstrasse 21, 70599 Stuttgart, Germany; phillip.mueter@uni-hohenheim.de (P.M.);

**Keywords:** plant-based milk alternatives, fouling, UHT-treatment, oat-drink processing, oat protein, protein concentration

## Abstract

Oat-based beverages are increasingly popular milk alternatives. However, the heat treatment required to ensure shelf stability is limited by rapid fouling formation on heated surfaces, reducing processing efficiency. Oat proteins, considered an important quality attribute of oat drinks, are suspected to play a key role in fouling initiation, but their specific contribution remains poorly understood. This study investigates the role of oat proteins in fouling formation during heat treatment on technical scale. Membrane filtration was applied and validated as sample preparation method for increasing the protein content. Fouling experiments were conducted using a previously validated fouling system with feed solutions containing different protein concentrations. Protein content was increased by filtration using 0.1, 0.8 and 1.4 µm ceramic membranes, yielding retentates with 10–21 g·100 g^−1^ on a dry matter basis, and further enriched to >40 g·100 g^−1^ through diafiltration. Fouling experiments (140 °C, 60 min) revealed a dependence of fouling formation on protein content in the feed solution. Fouling deposits were negligible at low protein concentrations (<2.5 g·100 g^−1^), increased markedly between 8 and 14 g·100 g^−1^, and reached a plateau at higher protein levels. Using oat supernatant or retentates, the protein content in the fouling correlated linearly with the protein content in the feed solution (*R*^2^ = 0.98) but did not exceed ~25 g·100 g^−1^, resulting in predominantly carbohydrate-based deposits. In contrast, diafiltered protein-enriched feed solutions produced larger, protein-dominated deposits. A conceptual model describing feed-dependent fouling mechanisms is proposed.

## 1. Introduction

Oat-based beverages are popular alternatives to cow’s milk, serving individuals with dietary restrictions like lactose intolerance or milk protein allergies, and those adhering to vegan diets [1,2]. The market has seen substantial growth and is projected to expand further due to ongoing innovation and product diversification [3,4]. To ensure microbial stability, oat drinks are typically subjected to thermal processing like ultra-high-temperature (UHT) treatment [5,6,7]. However, rapid fouling formation during thermal treatment has been reported, significantly limiting processing efficiency [8,9].

Fouling during the processing of liquid food systems results from the deposition of matrix components on heated surfaces [10,11]. While general fouling mechanisms in food processing are well-documented, the specific heat-induced interactions and deposition kinetics of oat-derived components—such as β-glucans, proteins, and starches—remain insufficiently understood [12]. Recent studies have applied advanced analytical techniques to monitor fouling in plant-based milk alternatives, but a deeper understanding of how variations in oat varietals, pre-processing methods, and specific UHT parameters influence the composition and adherence of fouling layers is still required [8,13]. Moreover, the effectiveness of different cleaning strategies tailored to the specific fouling characteristics of oat-based beverages needs more comprehensive investigation. Addressing these gaps through targeted research will be crucial for optimizing the thermal processing of oat-based drinks, ensuring consistent quality, and enhancing production efficiency in this growing market segment.

Among the oat beverage constituents, proteins are suggested to play a key role in fouling formation. Previous studies have hypothesized that, analogous to whey protein-induced fouling in milk processing, denaturation of oat protein could potentially destabilize the food matrix and govern the initiation and growth of fouling layers in oat drink heating [7,8,14]. Despite these indications, protein-specific contribution to fouling remains poorly understood. In their study about heat-induced fouling of oat drinks, Müter and Hinrichs [12] showed that, in addition to carbohydrates, proteins account for a major fraction of oat drink fouling deposits. Moreover, oat protein is not only relevant due to its potential role in fouling formation but is also widely regarded as a positively perceived plant protein and an important quality attribute of oat-based beverages [4,15].

Addressing this knowledge gap, the present study aims to systematically investigate the impact of protein on fouling formation during the heating of oat drinks. In the first step, cross-flow membrane filtration was employed to produce protein-enriched oat drinks, and the suitability of the sample preparation method was validated. Ceramic membranes with different nominal pore sizes were evaluated, and diafiltration was applied to increase protein purity. In the second and main step, these protein-enriched products were used to assess the effect of protein content on fouling formation. Fouling experiments were conducted using a self-designed, technical-scale fouling system.

## 2. Materials and Methods

### 2.1. Production of Oat Supernatant

For the production of the oat dispersion, 12.5 g·100 g^−1^ whole grain oat flour (Rubin Mühle GmbH, Lahr, Germany) was dispersed in demineralized water using a Robomix emulsifier (Model AU-L60, Algor System/La Nuovagel, Ottobrunn, Germany) at maximum speed for 5 min. Using 1 M NaOH, the pH was adjusted to 7.0. Starch was hydrolysed by adding 0.2% (*w*/*w*) of bacterial α-amylase (Delvo^®^Plant ALT, DSM Food Specialities B.V., Delft, The Netherlands). Enzymatic treatment was done in a tank at 60 °C for 120 min under continuous stirring. The oat dispersion was then decanted using a decanter centrifuge (Model CA 150-01-33, GEA Westfalia, Oelde, Germany) operated at maximum speed (6700 rpm, equivalent to 3760× *g*) and a differential speed of 3 rpm. Decanting was performed at 20 °C and a mass flow rate of 140 kg·h^−1^. The clarified product obtained after decanting is referred to as the (oat) supernatant.

### 2.2. Concentration of Oat Supernatant

The concentration of the protein content of the oat supernatant was carried out using a cross-flow membrane filtration (MF) unit (Model TFF, Pall GmbH, Dreieich, Germany). Because membrane-based concentration of oat protein is a novel approach, three ceramic membranes with different nominal pore sizes of 0.1, 0.8 and 1.4 µm were evaluated. All membranes had an effective area of 1.69 m^2^ and a channel diameter of 4 mm (M-7P1940 Membralox^®^ Module, Pall Exekia, Bazet, France).

Membrane selection was based on prior experiments indicating that oat protein concentration is feasible within this pore-size range. MF was performed at 50 °C with a transmembrane pressure (TMP) of 0.1 MPa. Before starting the concentration, the filtration was operated in equilibrium mode (i.e., the permeate was recirculated into the feed) for 10 min to stabilize parameters. Following concentration, the retentate was cooled immediately to 6 °C until further processing. If not used for fouling experiments, the permeate was discharged after taking a sample for analysis. For all filtration runs, the feed mass was 102 ± 5 kg, and filtration was continued until a retentate mass of 19 ± 2 kg was reached, corresponding to a volumetric concentration factor (VCF) of ~5.

The transmission (permeation) of a certain component was characterized by the transmission factor (*Tr_i_*). In the present study, the filtration was applied to concentrate the protein. Therefore, the protein-specific factor *Tr_p_*, calculated according to Equation (1), was used:

(1) $${Tr}_{p}=c_{p,permeate}/c_{p,retentate}$$

with $c_{p,permeate}$ representing the protein concentration in the permeate in g·100 g^−1^ and $c_{p,retentate}$ representing the protein concentration in the retentate in g·100 g^−1^.

### 2.3. Diafiltration of Oat Retentate

Protein purity in the retentate was enhanced by operating the filtration unit in diafiltration mode. The retentate obtained in the initial filtration step (Section 2.2) was diluted 1:1 (*w*/*w*) with demineralized water before the filtration procedure was repeated until the cumulative permeate mass equalled the mass of water added for dilution. In this study, four diafiltration cycles were performed. This stepwise process enabled the progressive removal of low-molecular-weight solutes (e.g., sugars and salts) through the membrane while retaining and concentrating the protein fraction in the retentate. The final retentate obtained after four diafiltration cycles was collected for further processing and analysis. To distinguish it from the regular retentates, the product obtained by diafiltration is hereafter referred to as the Dia-retentate.

### 2.4. Viscosity Adjustment by Xanthan Gum Addition

**Figure A1:**
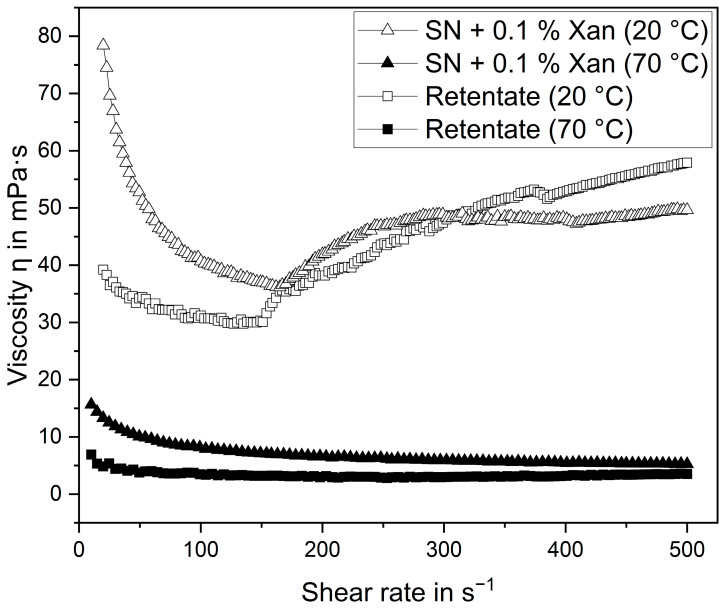
Comparative viscosity values as a function of shear rate for oat retentate (dry matter: 15.4 ± 0.1 g·100 g^−1^, protein in dry matter: 20.9 ± 0.3 g·100 g^−1^) and oat supernatant with 0.1% (*w*/*w*) Xanthan gum (Xan) measured at 20 and 70 °C. Standard deviations (*n* = 3) were low and are therefore not shown in the figure for improved readability.

In addition to increasing the dry matter and protein content, the concentration of the supernatant also resulted in higher viscosities in the obtained retentates. To distinguish viscosity effects from particle concentration effects, the bulk viscosity of supernatant was increased by adding Xanthan gum (ConGum XEH, Condio GmbH, Werder, Germany). Viscosity measurements (Appendix A, Figure A1) showed that the addition of 0.1 g·100 g^−1^ to supernatant resulted in viscosities comparable to those of the retentate samples with the highest protein content (protein in dry matter: 20.9 ± 0.3 g·100 g^−1^). The viscosity-increased supernatant samples are hereafter referred to as Xan-supernatant.

### 2.5. Fouling Experiments

Fouling experiments were conducted using a self-designed technical-scale test system (University of Hohenheim, Stuttgart, Germany), as described and validated by Müter and Hinrichs [12]. The system was specifically developed to simulate and accelerate fouling phenomena under controlled and reproducible conditions. The plant allows adjustment of critical process parameters known to promote fouling, including elevated temperature gradients, reduced product flow velocities, and the presence of entrained gas bubbles. By modulating these conditions, the setup enables rapid and consistent formation of fouling layers, thereby facilitating systematic investigation of fouling mechanisms.

Products with different protein content, produced as described in Section 2.1, Section 2.2, Section 2.3 and Section 2.4, were used as feed solutions for standardized fouling experiments to assess the impact of the protein content on fouling formation. For clarity, the different product groups are summarized in Table 1. The experimental procedure, including a detailed overview of the hydraulic parameters and technical details of the test section, has been described by Müter and Hinrichs [12]. In short, the product in the fouling system is preheated to 70 °C before entering the rectangular fouling test chamber. The temperature of the electrical heating plate in the fouling test chamber was set to 140 °C. For each fouling experiment, 15 kg of product were used. The system was operated in recirculation mode at a flow rate of 100 kg·h^−1^. The operation time per experiment was 60 min. The parameters were selected based on previous experiments with oat supernatant [12]. To isolate the effect of protein content on fouling formation, the temperature-time profile was kept constant in the present study.

### 2.6. Chemical Analysis

The dry matter content of all liquid samples was determined using an infrared moisture analyzer (Model MA 30, Sartorius GmbH, Göttingen, Germany). Approximately 3.0 ± 0.1 g of each sample was evenly distributed on a glass fiber filter paper and heated to 105 °C until a constant mass was achieved. Solid fouling samples were freeze-dried to obtain homogeneous powdered material for subsequent analyses. The dry matter content of the freeze-dried material was determined gravimetrically during the drying process. Samples were frozen at −40 °C and lyophilized under vacuum (0.1 mbar) using a pilot-scale freeze dryer (Model Sublimator 15, Zirbus Technology GmbH, Bad Grund, Germany). Following lyophilization, the dried samples were finely ground using a mortar and pestle to ensure uniformity. For reference, the dry matter content of the fouling samples was also determined using the drying oven method according to ISO 5534|IDF 4:2004 [16], with no significant deviation between methods. The total mineral content was determined according to the gravimetric method ASU L 06.00-4 [17].

Protein content was analyzed by determining the total nitrogen content using a combustion-type nitrogen analyzer (Dumatherm N Pro, C. Gerhardt GmbH & Co. KG, Königswinter, Germany), following the Dumas combustion principle in accordance with ISO 14891|IDF 185:2002 [18]. The nitrogen values obtained were converted to protein content using an oat-specific conversion factor of 5.36 [19].

### 2.7. Estimation of Protein Particle Size

The minimal size of a protein can be estimated from its molecular mass by assuming a spherical shape for globular proteins. Using reasonable assumptions and a partial specific volume $v_{P}$ of 0.73 cm^3^·g^−1^, Erickson [20] proposed that the minimal diameter of a protein molecule can be calculated according to Equations (2) and (3):

(2) $$V=\frac{v_{P}}{N_{A}}\cdot Mw$$

(3) $$d_{min}=2{\cdot (3V/4\pi )}^{1/3}$$

with *V*: the particle volume, $v_{P}$ the partial specific volume, Mw: the molecular weight, $N_{A}$: the Avogadro constant, and *d_min_*: the minimal particle diameter.

Applying this approach to the dominating 12S globulin in oat (Mw = 54 to 60 kDa, Boukid [21]) results in a minimal particle diameter of 5.0 to 5.2 nm.

### 2.8. Sodium Dodecyl Sulphate–Polyacrylamide Gel Electrophoresis (SDS-PAGE)

SDS-PAGE under non-reducing conditions was performed for protein separation and fraction identification of the liquid samples. The samples were standardized to a protein content of 0.2% (*w*/*w*) and mixed with a sample buffer prepared according to Laemmli [22]. A volume of 15 µL of each sample was loaded onto the gel (Mini-PROTEAN TGX Stain-free gels 4–20%, 10-well comb, 50 µL, Bio-Rad Laboratories, Feldkirchen, Germany), along with a molecular-weight standard (10–250 kDa, Precision plus protein standards, Bio-Rad Laboratories, Feldkirchen, Germany). Electrophoresis was conducted at 200 V for approximately 30 min. Proteins were stained using ROTI^®^Blue staining solution (Carl Roth GmbH & Co. KG, Karlsruhe, Germany) for 24 h, followed by destaining in demineralized water for 24 h. Gels were evaluated qualitatively by image analysis.

### 2.9. Rheology

Apparent viscosity of the liquid samples was measured using a rotational rheometer (Model MCR 502, Anton Paar GmbH, Ostfildern, Germany) with a concentric cylinder measuring geometry (CC27, cup radius = 14 mm) according to DIN 53019. Measurements were conducted at 20 °C to evaluate ambient viscosity and at 70 °C to simulate high temperature conditions. A volume of 25 mL of sample was added to the cup and equilibrated for 5 min prior to the experiment. The shear rate was increased linearly from 10 to 500 s^−1^ over 5 min.

### 2.10. Particle Size Distribution

The particle size distribution of the liquid samples was measured at room temperature using a static light scattering particle size analyzer (Type LS 13 320, Beckman Coulter, Inc., Brea, CA, USA). Each sample (200 µL) was pipetted into the measurement cell containing demineralized water as the dispersion medium. The refractive index was set to 1.47 for oat protein and 1.33 for the solvent (water). The polarization intensity differential scattering (PIDS) signal for all measurements ranged between 40 and 60%, and obscuration levels were maintained between 3 and 5% to ensure optimal measurement conditions. For each sample, triplicate measurements were performed, with each measurement representing the average of three internal runs, resulting in a total of nine data points per sample. The particle size is described as volume-based *d*_90_ (*d*_90,3_), defined as the diameter below which 90% of the total particle volume is found.

### 2.11. Confocal Laser Scanning Microscopy (CLSM)

The protein structure of liquid samples was examined using a confocal laser scanning microscope (Model LSM 900, Carl Zeiss Microscopy GmbH, Jena, Germany). In total, 300 µL of 0.2% (*w*/*v*) Nile Blue solution (N5632, Th. Geyer, Renningen, Germany) was mixed with 200 µL of the sample at room temperature. The stained sample was placed in a cover glass-bottomed imaging dish (Imaging Dish CG 1.5, Miltenyi Biotec GmbH, Germany). Microscopy was performed at 63× magnification. Nile Blue was excited using a 640 nm laser to visualize the protein phase. A series of approximately 30 images was captured along the z-axis within the visible sample depth and subsequently combined into a single image using Zen Blue software (LSM 900, Carl Zeiss Microscopy GmbH, Jena, Germany).

### 2.12. Statistical Analysis

All analyses were conducted in triplicate. For all analyses, the arithmetic means and the standard deviation were calculated. Statistical significance was tested using one-way ANOVA at a significance level of *p* < 0.05, followed by Tukey´s post hoc test for pairwise comparison. For experiments conducted in duplicate, statistical significance was evaluated using a two-tailed t-test assuming equal variances. Statistical analysis was performed using OriginPro 2024 (OriginLab Corporation, Northampton, MA, USA).

## 3. Results and Discussion

### 3.1. Evaluation of Sample Preparation by Microfiltration

As sample preparation substantially affected the quality and reproducibility of the subsequent fouling experiments, the procedure was systematically evaluated. Given the limited information available on comparable sample preparation approaches for oat drinks at this scale, the filtration process was analyzed in detail to assess its influence on the interpretation of the fouling experiments discussed in Section 3.2. Microfiltration was carried out using ceramic membranes with nominal pore sizes of 0.1, 0.8 and 1.4 µm to obtain protein-enriched retentates. The feed material, the self-produced oat supernatant, showed high reproducibility, with dry matters of 9.8 ± 0.5 g·100 g^−1^ and protein contents of 0.8 ± 0.1 g·100 g^−1^ (*n* = 9).

#### 3.1.1. Protein Transmission

Protein transmission increased from 8.0 to 16.0% with increasing membrane pore size; however, these differences were not statistically significant (Table 2). Protein transmission was calculated based on a composite permeate sample. For all experiments, the permeate protein content remained constant and was therefore independent of the sampling time point.

The protein transmission through all tested membranes was lower than anticipated based on the molecular weight of oat proteins and the corresponding diameters calculated in Section 2.7. Furthermore, it is known that, for example, milk protein, including casein micelles, which are considerably larger than oat proteins, can permeate microfiltration membranes [10]. To better understand the high retention of oat proteins observed for all tested membranes, the structural characteristics of oat proteins must be considered. It is reported that 12S globulins undergo a two-stage self-assembly process in aqueous solutions, initially forming trimers that subsequently assemble into hexamers [23]. Depending on the analytical approach, these hexameric structures with a molecular weight of approximately 320 kDa have been described as ellipsoidal particles with diameters of around 12 nm or droplet-like structures with dimensions of approximately 12 × 8.5 nm [23,24,25]. In their review on oat globulin, McLauchlan et al. [23] summarize that the hydrodynamic diameter of oat globulin can span wide ranges, even higher than 100 nm. Beyond protein self-assembly, interactions with other matrix components, e.g., the formation of aggregates with β-glucan, further influence the effective size of oat proteins [26]. In summary, the effective protein particle size is strongly influenced by the surrounding liquid matrix and physicochemical conditions, which could contribute to the high protein retention observed in this study [23,25,27].

**Figure 1:**
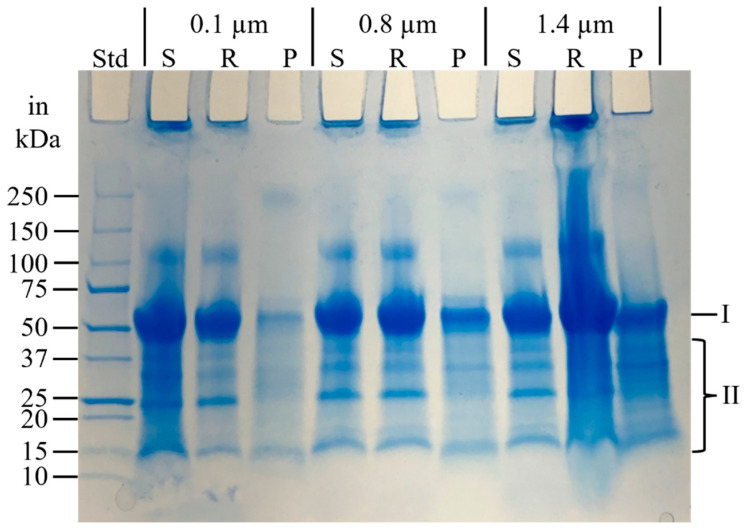
SDS-PAGE gel of oat supernatant, retentate and permeate under non-reducing conditions. Std: molecular-weight standard, S: supernatant, R: retentate, P: permeate. Protein bands were assigned as follows: I: 12S globulin, 7S globulin; II: albumins, 3S globulin, α- and β- subunits of 12S globulin; these are based on works by Burgess et al. [29], Ma and Harwalkar [30], and McLauchlan et al. [26].

SDS-PAGE analysis of the supernatant samples (Figure 1) revealed several protein bands characteristic of oat drinks under the neutral extraction conditions applied in this study, consistent with literature reports on oat protein composition [23]. The most intense band observed at approximately 50 kDa can be attributed to the predominant oat globulin (12S globulin) in its monomeric form [23,28]. In the context of this study, the comparability of the identified protein bands in the supernatants and corresponding retentates for all three membranes is of particular importance. The identical protein band patterns indicate that the selected membranes generally fulfilled the requirements of this study, as all protein fractions present in the supernatants, including those with lower molecular weights, were effectively retained and consequently concentrated in the retentates. The permeation of both albumins and globulins was further confirmed by semi-quantitative HPLC analysis. This is desirable, as the retentates used for the subsequent fouling experiments should contain all proteins naturally occurring in the supernatant, but at higher concentrations. Although no significant differences in protein transmission were observed among the three membranes (Table 2) and the protein content was identical in all samples, the band intensities in the permeates increased with increasing pore size (Figure 1). This could reflect minor differences in the molecular-weight distribution of the transmitted proteins, with proteins passing through larger-pore membranes being more readily visualized as distinct bands. However, in the context of the present study, the critical observation is that all protein fractions remained present in the retentates, as discussed above.

**Figure A2:**
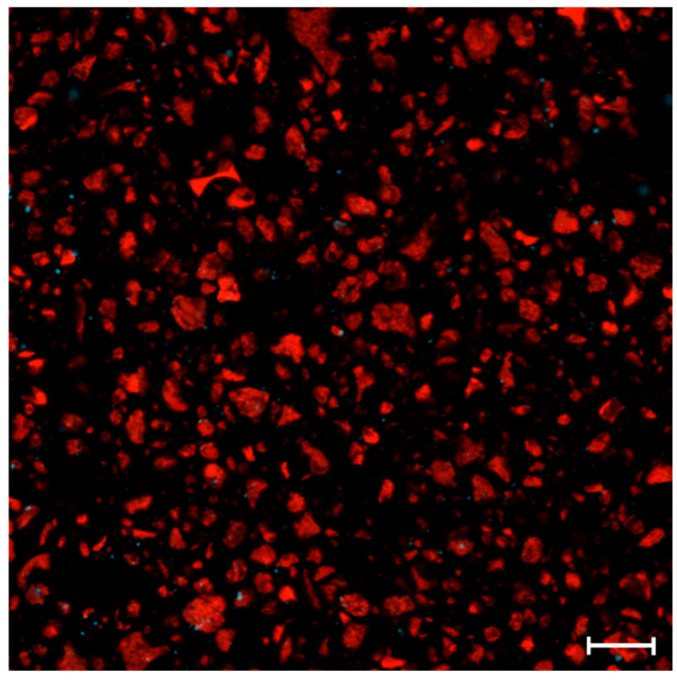
CLSM images (63× magnification) of oat supernatant. Nile Blue was excited to visualize the protein phase in red. The scale bar is 10 µm.

Particle size analysis of the oat supernatant used in the present study by dynamic light scattering resulted in a *d*_90,3_ of 3.4 ± 0.4 µm. These values are comparable to those reported in other studies on technical-scale oat supernatant production [12,31]. The results indicate the presence of particles substantially larger than the pore sizes of the used membranes. Although these particles can also consist of carbohydrates or dietary fiber rather than protein, CLSM images (Appendix A, Figure A2) nevertheless suggest the presence of protein aggregates exceeding 1 µm, consistent with McLauchlan et al. [26]. Overall, the results indicate that, at least for a fraction of the proteins, particle sizes are larger than expected based on molecular weight.

In addition to the consideration of the protein particle size, it should be noted that efficient protein-permeation cannot be assumed solely based on the protein size being smaller than the nominal pore sizes, as proteins generally require pore diameters several-fold larger than their hydrodynamic size to overcome steric and hydrodynamic hindrance effects as well as membrane fouling influences and instantaneous gel-layer formation [32]. Such discrepancies between size-based predictions and experimental transmission data have been reported previously, for example, for milk proteins [33,34]. Comparable data for the filtration of oat supernatant at pilot-scale working with cross-flow membrane systems are lacking.

#### 3.1.2. Diafiltration of Oat Retentate

Diafiltration was applied (*n* = 1; 0.8 µm membrane) to increase protein purity in the retentate. After four diafiltration cycles, low-molecular-weight constituents were effectively washed out, resulting in an increase in the protein in dry matter from 8.8 ± 0.5 g·100 g^−1^ in the supernatant to 42.0 ± 1.2 g·100 g^−1^ in the final retentate (Appendix A, Table A1). The dry matter content in the permeate of the final filtration cycle was 0.3 ± 0.1 g·100 g^−1^, indicating that removable particles had been separated. Using ultrafiltration with a molecular-weight cut-off of 10 kDa in diafiltration mode, Immonen et al. [28] achieved a protein concentration of approximately 39 g·100 g^−1^ (protein factor corrected to 5.36). This comparison suggests that the larger pore size of the membrane used in the present study did not negatively affect protein retention. This is consistent with the low protein transmission observed for the 0.8 µm membrane as discussed above. These results suggest that even at higher protein contents in the oat retentate and thus reduced matrix effects (e.g., due to lower concentration of carbohydrates), protein transmission is not increased compared to oat supernatant. In addition, SDS-PAGE gels of the dia-retentate again revealed protein bands similar to those previously observed for the different concentrates (Figure 1).

#### 3.1.3. General Discussion

While filtration is a commonly applied technique for protein concentration, it is noteworthy that in the case of oat protein, membranes with pore sizes substantially larger than the reported protein size still exhibit high protein retention. Although possible explanations were discussed in this chapter, further investigation is required.

Nonetheless, it should be emphasized that the primary objective of the microfiltration experiments in this study was to generate protein-enriched retentates for subsequent fouling investigations. Accordingly, membrane selection and evaluation of concentration performance served the purpose of preparing suitable feed materials for controlled fouling experiments. In this regard, protein retention was considered sufficiently high, and the major protein fractions present in oat supernatant were successfully concentrated using all three membranes.

Among the tested membranes, the 0.8 µm variant proved most suitable for generating a concentrated oat dispersion with balanced protein content and acceptable filtration performance. Therefore, the 0.8 µm membrane was selected to produce the retentates used for the subsequent fouling experiments.

Further optimization of the filtration process can be possible, as has been demonstrated for milk protein fractionation [32,34], but this was beyond the scope of the present work. Nevertheless, comparative analysis provided valuable insights into the influence of membrane characteristics on oat protein enrichment. These findings are not only interesting when filtration is used for sample preparation, as in our study, but also particularly relevant for potential technical-scale applications of oat drink filtration, such as the use of microfiltration for bacterial reduction or to produce protein-enriched oat drinks.

### 3.2. Fouling Experiments

Fouling experiments (100 kg·h^−1^, 140 °C, 60 min) were conducted with the different product groups. The results for fouling mass, dry matter, and protein content are summarized in Table 3. No fouling occurred with permeate as the feed solution. Using supernatant, retentate, or Xan-supernatant as feed resulted in high fouling dry matter contents of more than 90 g·100 g^−1^, whereas the fouling obtained from Dia-retentate had a significantly lower dry matter of approximately 46 g·100 g^−1^. Protein in fouling dry matter was approximately 12 g·100 g^−1^ using supernatant as feed solution and increased significantly with protein content in the retentates, reaching 17–27 g·100 g^−1^. The Dia-retentate resulted in the highest protein content in the fouling layer (>80 g·100 g^−1^), while Xan-supernatant fouling showed the lowest (<10 g·100 g^−1^, not significantly different from supernatant).

Fouling mass increased from approximately 19 mg·cm^2^ in the supernatant experiments to 30–40 mg·cm^2^ for the different retentates, with no significant differences within the retentate group. Fouling mass in the Dia-retentate was the highest, reaching 101 ± 13 mg·cm^2^. Fouling mass of the Xan-supernatant experiments (18.4 ± 4.4 mg·cm^2^) did not differ significantly from the supernatant experiments. Regarding composition, the combined contents of minerals, fat, and β-glucan were below 10 g·100 g^−1^ in all fouling samples. None of these ingredients showed scaling under the tested conditions. Although no direct carbohydrate analysis was performed, this indicates that, apart from protein, carbohydrates constituted the predominant fraction of the remaining fouling dry matter.

With respect to minerals, it should be noted that although minerals such as calcium are generally considered relevant for fouling during heat treatment and are often added to commercial oat drinks [35], no minerals were added in this study to isolate the effect of protein content using non-fortified supernatant. Therefore, mineral-induced fouling was considered negligible under the conditions investigated, but its effect on fouling should be addressed in future studies.

These quantitative observations are consistent with the visual appearance: Fouling layers formed from supernatants, retentates and Xan-supernatant exhibited a dense, dry and crust-like structure (Table 4) and adhered strongly to the surface. These fouling layers showed an intense brown coloration. In contrast, the fouling layers derived from Dia-retentate appeared grayish, with noticeably reduced presence of brown or dark discoloration (Table 4). Texture and adherence were visibly voluminous, moist, and exhibited a slimy consistency. It was loosely associated with the stainless-steel surface of the test section and could be removed easily.

The absence of fouling in the permeate experiments suggests that at low protein concentrations, the availability of thermally labile protein is insufficient to initiate deposit nucleation [36]. This finding is consistent with the findings of Da Matos et al. [8], who suggested that protein denaturation initiates fouling formation during oat drink heating. The increase in fouling mass between the supernatant and retentate product groups is likely attributable to increased protein availability [37]. In addition, the higher dry matter content, and thus the higher concentration of potential foulants at the heated surface, can further promote fouling formation. Within the retentate group, the fouling mass did not increase with increasing protein content (Table 3). This is potentially due to surface saturation effects [38]. A higher protein content can promote faster fouling formation, and once a compact fouling layer is established, it can act as a thermal and physical barrier, thereby reducing the net deposition rate [39].

The Dia-retentate experiments exhibited more than a twofold increase in fouling mass deposition, showing that high protein purity and thus lower availability of carbohydrates promote fouling formation. In contrast, the fouling experiments using Xan-supernatant showed that even though increasing bulk viscosity changes the flow resistance and the macroscopic boundary-layer thickness [40], it does not increase the concentration of proteins capable of undergoing heat-induced structural conversion at the wall. It produced markedly less fouling mass and a lower proportion of protein in deposits than the (Dia-)retentates. Overall, these results indicate that protein concentration is the dominant factor influencing deposit formation.

To summarize the effects of the feed solution composition on fouling formation, a schematic illustration was proposed (Table 5). While additional investigation of the underlying molecular interactions is required, the model illustrates the fouling mechanisms as suggested based on the results described in this study. Fouling is likely initiated by heat-induced unfolding and subsequent reactions of oat protein, mainly the predominant 12S/7S globulins (see Section 3.1), which are known to denature and aggregate at temperatures above 100 °C [30,41]. The reaction of the unfolded protein includes protein–protein and protein–carbohydrate interactions, which will be further discussed below.

When interpreting the fouling results, it should be noted that all experiments were conducted under fixed operating conditions (mass flow rate, heating temperature, and fouling duration). Although these parameters are generally known to influence fouling formation and deposit composition, a previous study by the authors demonstrated that, for oat supernatant and using the fouling system applied in the present work, extended fouling durations affected the amount of deposited material but not its composition [12]. Furthermore, the same study showed that fouling of oat supernatant occurred only at temperatures above 120 °C, thereby limiting the relevant process window. Together, these findings suggest that the fouling mechanisms proposed in the present study are not strongly dependent on fouling duration within the investigated range and can therefore be applicable across a broader set of operation conditions within the relevant temperature regime.

**Figure 2:**
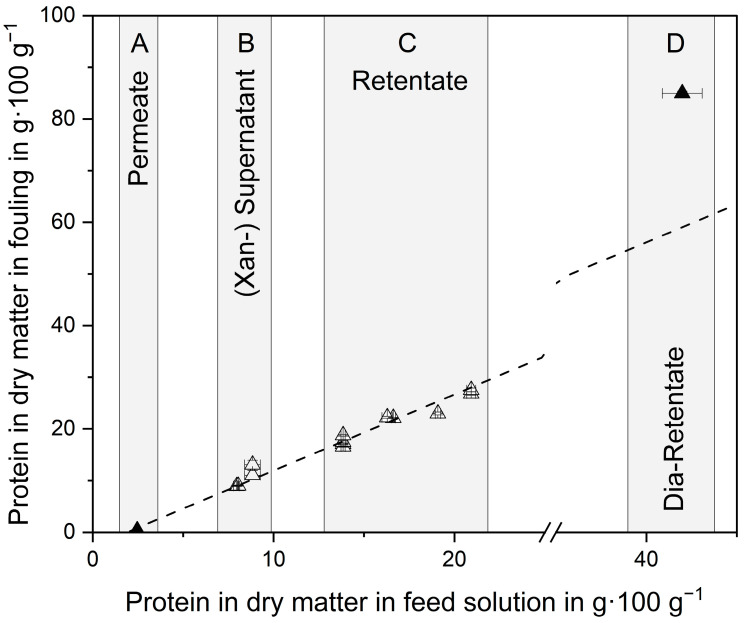
Graphical representation of the protein in dry matter data presented in Table 3. Gray shaded areas describe the different product groups used as feed solutions: A: experiments with permeate, B: experiments with supernatant and Xan-supernatant, C: experiments with retentate, D: experiments with Dia-retentate. The dotted line represents a linear regression fitted to data from areas B and C (*R*^2^ = 0.98) and extended across the entire plot. Data points in areas A and D are shown as closed symbols to indicate that they are not included in the regression. For some data points, error bars (±standard deviation) are smaller than the symbol size and therefore not visible.

With respect to the fouling composition, the results (Table 3) showed that the feed solution composition influenced the protein content of the fouling layer. To illustrate the effect of protein concentration in the feed solution on the protein content of the fouling layer, the relationship is presented graphically in Figure 2. For supernatants and retentates, a strong linear relationship (*R*^2^ = 0.98) was observed, indicating the feed protein-content dependency of oat fouling during heat treatment. However, while the protein content of the fouling layer increased, it did not exceed 30 g·100 g^−1^, and consequently, the fouling remained carbohydrate-dominated. While this is the first study to investigate different protein contents in the feed solution, a previous study by the authors confirmed that oat drink fouling is carbohydrate-based [12]. Similar findings have been reported for soy drink fouling [42].

Overall, it can be concluded that in mixed composition feeds containing less than 25 g·100 g^−1^ protein in feed dry matter, the protein content of the fouling shows a positive relationship with protein content of the feed, although carbohydrates account for the major fraction of the deposit. Feed protein content is also positively related to fouling mass; however, saturation effects are seen in the fouling setup used in this study when the protein content reached approximately 15 g·100 g^−1^. It is suggested that protein–carbohydrate interactions (e.g., via Maillard reactions or co-precipitation with degraded polysaccharides) lead to the formation of a fouling layer that is less dense in protein, mechanically firm and dry and enriched in thermally modified carbohydrates [43,44]. The brown coloration previously presented in Table 4 further indicates the occurrence of Maillard reactions [44].

The fouling composition changed towards a strongly protein-dominated fouling when Dia-retentate was used as the feed solution. The high protein content of the fouling of more than 80 g·100 g^−1^ suggests that the fouling mechanism was protein-specific, with minimal incorporation of other macromolecules. The coloration of the fouling, as shown in Table 4, underlines that the Maillard reaction, and thus protein–carbohydrate interactions, were less relevant and the fouling mechanism shifted towards direct protein aggregation at the heated surface. This compositional difference in protein-based fouling is further evidenced by the protein contents of the fouling layers of the Dia-retentate experiments, whose values clearly deviate from the linear regression between protein content in feed solution and fouling layer observed for supernatants and retentates (Figure 2, B–D). This observation is consistent with findings from dairy systems, showing that once proteins dominate the deposit layer, their deposition is largely self-sustaining. Denatured proteins form a cohesive matrix that entraps additional protein aggregates but resists incorporation of soluble carbohydrates or minerals [9,10]. Moreover, studies on dairy matrices have shown that feed containing higher relative amounts of carbohydrates resulted in deposits that were less protein-dense, more open in structure, and lower in total mass, while protein-enriched feeds devoid of sugars generated cohesive protein layers prone to heavy accumulation [43]. These findings are in agreement with the results of the present study.

In general, these observations reinforce the idea that the protein-to-carbohydrate profile can alter deposit morphology in ways that influence not only fouling, but also cleaning behavior. Carbohydrate–protein interactions, whether through physical mixing or Maillard-type conjugation, appear central to determining not only how much fouling forms, but also the physical character of the deposit. From a process control perspective, these findings underscore that protein purity, rather than total solids content, is the more reliable predictor of protein-dominated fouling risk in oat drink heating processes. While high sugar and polysaccharide content can moderate protein fraction in deposits by promoting mixed-material fouling layers, removal of these components—whether intentionally through diafiltration or unintentionally through upstream separation steps—can produce protein-rich deposits. This has important implications for both process design and cleaning-in-place (CIP) procedures, as effective cleaning depends on selecting and sequencing cleaning agents according to the morphology and composition of the fouling layer [9,44].

## 4. Conclusions

This study investigated protein-mediated fouling of oat-based systems under UHT surface conditions with the main objective of determining how feed protein concentration controls fouling mass, composition and morphology. Protein content in the oat supernatant was successfully increased using 0.1, 0.8 or 1.4 µm membranes, reaching values of 13 to 21 g·100 g^−1^ on a dry matter basis. Through diafiltration, protein content in dry matter could be increased further to values exceeding 40 g·100 g^−1^. The suitability of membrane filtration as a sample preparation method was successfully validated.

Standardized fouling experiments conducted at a heating temperature of 140 °C for 60 min demonstrated dependency of fouling formation on protein content of the feed solution: Fouling deposit mass was negligible at low protein concentrations (<2.5 g·100 g^−1^ on dry matter basis), increased sharply with increasing protein in dry matter content from 8 to 14 g·100 g^−1^, and reached a plateau when protein content in the retentates was increased further. From a composition perspective, protein content of the fouling deposits showed a clear linear correlation (*R*^2^ = 0.98) to the protein content in the feed solution, but did not exceed approximately 25 g·100 g^−1^. As a result, fouling layers formed from supernatant or retentates resulted in carbohydrate-dominated fouling layers that appeared brown-colored, dry, and strongly adhered to the surface.

In contrast, diafiltered, protein-enriched feed solutions resulted in larger, protein-dominant deposits, which exhibited a wet, voluminous and weakly adhered structure. Viscosity-matched controls (supernatant supplemented with Xanthan gum) produced significantly less fouling, confirming that protein availability and composition, not bulk viscosity, are the dominant drivers of heat-induced fouling. A model was developed to illustrate the feed-dependency of the fouling mechanisms as presented in this work.

While the findings described in this study advance the general understanding of the role of oat protein in heat-induced fouling formation, the molecular mechanisms underlying protein denaturation, aggregation, and interfacial interactions remain insufficiently described. Further research on protein behavior is required to enable predictive fouling models and the development of effective fouling mitigation strategies. In addition, fouling experiments should be extended to investigate time- and temperature-dependent changes in fouling formation as well as cleaning behavior.

## Figures and Tables

**Table 1:** Overview of the different product groups used as feed solutions for the fouling experiments and their approximate protein contents.

Product Group	Description	Protein Content in Dry Matter in g·100 g^−1^
Permeate	Permeate obtained from membrane filtration of supernatant produced as described in Section 2.2	~2.5
Supernatant	Regular oat supernatant produced according to Section 2.1	~8.5
Retentate	Retentates obtained from membrane filtration of supernatant, produced as described in Section 2.2. Retentates with different protein contents have been used.	10–21
Dia-Retentate	Dia-filtered retentate obtained from diafiltration of retentate produced as described in Section 2.3	~42
Xan-Supernatant	Viscosity-increased supernatant produced as described in Section 2.4	~8.5

**Table 2:** Protein transmission of the three tested membranes (*n* = 3). For all filtration runs, the volumetric concentration factor was ~5. Filtration was conducted at 50 °C and a transmembrane pressure of 0.1 MPa. Superscript letters indicate statistically significant (*p* < 0.05) differences. Values sharing one letter are not significantly different.

Membrane Nominal Pore Size	Protein Transmission *Tr_p_* in %
0.1 µm	8.0 ± 0.6 ^a^
0.8 µm	9.9 ± 2.4 ^a^
1.4 µm	16.0 ± 5.7 ^a^

**Table 3:** Dry matter and protein contents of the different feed solutions by product group, together with fouling dry matter, protein content and mass. *n*: number of individual fouling experiments. Different superscript letters indicate statistically significant (*p* < 0.05) differences within columns. Values sharing one letter are not significantly different. n.a.: not applicable as no fouling was detected.

Product Group	*n*	Feed Solution		Fouling		
		Dry Matter in g·100 g^−1^	Protein in Dry Matter in g·100 g^−1^	Dry Matter in g·100 g^−1^	Protein in Dry Matter in g·100 g^−1^	Mass in mg·cm^2^
Permeate	2	8.7 ± 0.1	2.5 ± 0.1	n.a.	n.a.	n.a.
Supernatant	3	10.1 ± 0.3	8.8 ± 0.5	90.4 ± 1.5 ^a^	12.1 ± 1.5 ^a^	18.8 ± 1.2 ^a^
Retentate	4	12.0 ± 0.1	13.8 ± 0.2	95.0 ± 1.1 ^b^	17.5 ± 1.0 ^b^	38.5 ± 2.6 ^b^
	2	12.7 ± 0.3	16.5 ± 0.3	93.1 ± 1.0 ^ab^	22.2 ± 0.1 ^c^	30.9 ± 0.3 ^c^
	2	14.7 ± 0.1	19.1 ± 0.2	94.0 ± 1.5 ^ab^	23.0 ± 0.2 ^d^	40.4 ± 2.1 ^b^
	3	15.4 ± 0.1	20.9 ± 0.3	93.1 ± 0.3 ^ab^	27.2 ± 0.6 ^e^	39.1 ± 0.5 ^b^
Dia-Retentate	2	9.0 ± 0.3	42.0 ± 1.2	46.1 ± 1.0 ^c^	85.0 ± 2.5 ^f^	101 ± 13 ^d^
Xan-Supernatant	2	10.3 ± 0.2	8.4 ± 0.2	93.1 ± 0.1 ^ab^	9.0 ± 0.1 ^a^	18.4 ± 4.4 ^a^

**Table 4:** Representative images of the fouling plates per product group. Flow direction from left to right. n.a.: not applicable as no fouling was detected.

Product Group	Representative Fouling Plate
Permeate	n.a.
Supernatant	^ 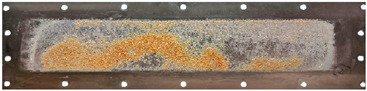 ^
Retentate	^ 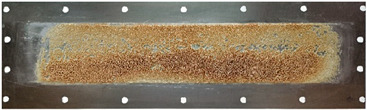 ^
Dia-Retentate	^ 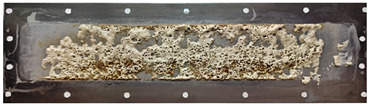 ^
Xan-Supernatant	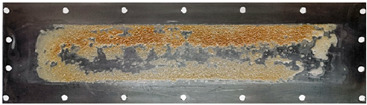

**Table 5:** Proposed simplified schematic representation of the feed solution composition and the fouling mechanism in the test section. The model is limited to the primary constituents of the feed solution, namely protein and carbohydrates. Fouling layer thicknesses are illustrative and not drawn to scale.

	Feed Solution	Fouling Test Section	Legend	
Permeate	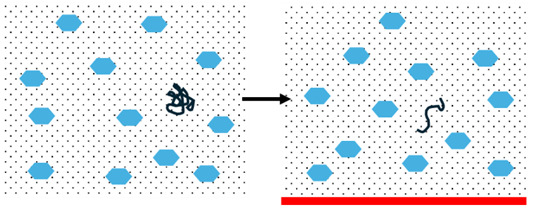		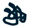	Native Protein
			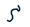	Unfolded Protein
			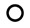	Reacted Protein
			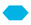	Sugar/Dextrin
			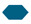	Reacted Sugar/Dextrin
Supernatant	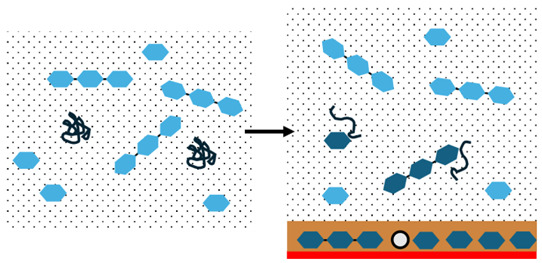		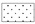	Oat Matrix
			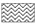	Viscous Oat Matrix
			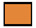	Carbohydrate-Dominated Fouling
			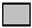	Protein-Dominated Fouling
			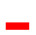	Hot Plate (140 °C)
Retentate	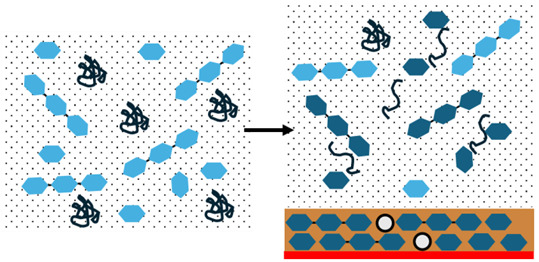			
Dia-Retentate	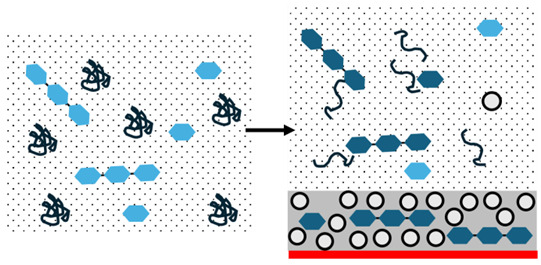			
	
	
	
	
	
Xan-Supernatant	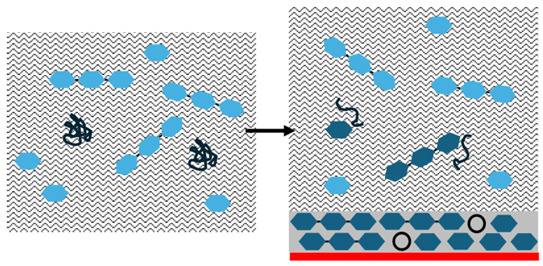			
	
	
	
	

## Data Availability

The original contributions presented in this study are included in the article. Further inquiries can be directed to the corresponding author.

## Abbreviations

The following abbreviations are used in this manuscript:

CIP	Cleaning-in-Place
CLSM	Confocal Laser Scanning Microscopy
Dia	Diafiltration
HPLC	High-Performance Liquid Chromatography
MF	Membrane Filtration
SDS-PAGE	Sodium Dodecyl Sulphate–Polyacrylamide Gel Electrophoresis
UHT	Ultra-High-Temperature
Xan	Xanthan Gum

## Appendix A

**Table A1:** Dry matter, protein in dry matter of the oat supernatant before filtration (cycle 0) and of the oat retentate and dia-retentate after each filtration cycle (cycles 1–5). From cycle 0 to 1, 156.1 kg of oat supernatant were concentrated to 20.7 kg oat retentate. In each diafiltration cycle, the concentrate from the previous filtration run was diluted with demineralized water at a ratio of 1:1 (*w*/*w*) prior to filtration. All filtration cycles were conducted at 50 °C using the 0.8 µm membrane. Different superscript letters indicate statistically significant (*p* < 0.05) differences within columns. Values sharing one letter are not significantly different.

Cycle	Dry Matter in g·100 g^−1^	Protein in Dry Matter in g·100 g^−1^
0 (Supernatant)	10.1 ± 0.5 ^a^	8.8 ± 0.5 ^a^
1 (Retentate)	14.7 ± 0.2 ^b^	26.8 ± 0.4 ^b^
2 (Dia-retentate cycle 1)	11.4 ± 0.3 ^c^	30.8 ± 0.7 ^c^
3 (Dia-retentate cycle 2)	9.7 ± 0.4 ^ad^	35.9 ± 1.2 ^d^
4 (Dia-retentate cycle 3)	9.2 ± 0.3 ^de^	41.5 ± 1.4 ^e^
5 (Dia-retentate cycle 4)	9.0 ± 0.3 ^e^	42.0 ± 1.2 ^e^
